# A brief bout of exercise in hypoxia reduces ventricular filling rate and stroke volume response during muscle metaboreflex activation

**DOI:** 10.1007/s00421-020-04435-0

**Published:** 2020-07-19

**Authors:** Gabriele Mulliri, Sara Magnani, Silvana Roberto, Fabio Sechi, Giovanna Ghiani, Gianmarco Sainas, Giorgio Nughedu, Seyed Alireza Hosseini Kakhak, Pier Paolo Bassareo, Antonio Crisafulli

**Affiliations:** 1 Sports Physiology Laboratory, Department of Medical Sciences and Public Health, University of Cagliari, Via Porcell 4, 09124 Cagliari, Italy; 2 International PhD in Innovation Sciences and Technologies, University of Cagliari, Cagliari, Italy; 3 Faculty of Physical Education and Sport Sciences, Hakim Sabzevari University, Sabzevar, Iran; 4 University College of Dublin, Mater Misericordiae University Teaching Hospital, Dublin, Ireland

**Keywords:** Blood pressure, Cardiac output, Cardiac pre-load, Myocardial contractility, Venous return

## Abstract

**Purpose:**

The hemodynamic consequences of exercise in hypoxia have not been completely investigated. The present investigation aimed at studying the hemodynamic effects of contemporary normobaric hypoxia and metaboreflex activation.

**Methods:**

Eleven physically active, healthy males (age 32.7 ± 7.2 years) completed a cardiopulmonary test on an electromagnetically braked cycle-ergometer to determine their maximum workload (*W*_max_). On separate days, participants performed two randomly assigned exercise sessions (3 minutes pedalling at 30% of *W*_max_): (1) one in normoxia (NORMO), and (2) one in normobaric hypoxia with FiO_2_ set to 13.5% (HYPO). After each session, the following protocol was randomly assigned: either (1) post-exercise muscle ischemia (PEMI) to study the metaboreflex, or (2) a control exercise recovery session, i.e., without metaboreflex activation. Hemodynamics were assessed with impedance cardiography.

**Results:**

The main result was that the HYPO session impaired the ventricular filling rate (measured as stroke volume/diastolic time) response during PEMI versus control condition in comparison to the NORMO test (31.33 ± 68.03 vs. 81.52 ± 49.23 ml·s^−1^,respectively, *p* = 0.003). This caused a reduction in the stroke volume response (1.45 ± 9.49 vs. 10.68 ± 8.21 ml, *p* = 0.020). As a consequence, cardiac output response was impaired during the HYPO test.

**Conclusions:**

The present investigation suggests that a brief exercise bout in hypoxia is capable of impairing cardiac filling rate as well as stroke volume during the metaboreflex. These results are in good accordance with recent findings showing that among hemodynamic modulators, ventricular filling is the most sensible variable to hypoxic stimuli.

## Introduction

Hypoxia triggers numerous adaptive mechanisms at cellular, tissue, and systemic level. At tissue level, it elicits local vasodilation mediated by nitric oxide (NO), which in turn induces vascular smooth cell relaxation (Bolotina et al. 1994; Marshall 2015). At systemic level, carotid and aortic chemoreceptor act as peripheral sensors of hypoxia and mediate the activation of the sympathetic nervous system (SNS), thus leading to an increase in heart rate (HR), cardiac output (CO), and pulmonary ventilation (*V*_E_) (Naeije et al. 2010; Stembridge et al 2015). The CO increment seems to be mainly HR-driven, as stroke volume (SV) has been reported to be unchanged (Talbot et al. 2005) or reduced (Stembridge et al. 2015) in hypoxic conditions.

During exercise bouts, neural mechanisms regulate the circulation on the basis of central and peripheral feedback (Amann et al. 2011; Nobrega et al. 2014). In one mechanism, the motor cortical areas reflexively activate the cardiovascular control centres located in the medulla oblongata, inducing an increase in HR and in mean blood pressure (MAP) proportional to motor drive. This mechanism, termed “central command”, produces its effects by increasing SNS activity and by withdrawing vagal tone. The resulting cardiovascular response is then finely adjusted on the basis of the feedback coming from periphery. Specifically, at muscle level, type III and IV nerve endings collect information about the mechano and metabolic status of the contracting muscle and convey this information to the cardiovascular control areas, thereby triggering a SNS-mediated reflex termed “exercise pressor reflex” (EPR). The metabolic part of the EPR is commonly termed “metaboreflex”, whereas the mechanical one is known as “mechanoreflex” (Crisafulli et al. 2015; Nobrega et al. 2014).

While the effects of hypoxia on cardiovascular variables during dynamic exercise have been extensively studied (Casey and Joyner 2012; Rowell and Blackmon 1987; Siebenmann and Lundby 2015; Wagner 2000), research dealing with the interaction between hypoxia and the neural regulation of cardiovascular activity is still scarce. Houssiere et al. (2005) investigated the effect of hypoxia and the metaboreflex elicited by post-exercise muscle ischemia (PEMI) method. This manoeuvre allows to isolate the metaboreflex from both central command and mechanoreflex activity. The authors concluded that hypoxia and metaboreflex exert a different effect on the cardiovascular and autonomic nervous system, with hypoxia showing a greater influence on HR and *V*_E_, whereas the metaboreflex is the main determinant of muscle sympathetic nerve activity and blood pressure. In a successive study, the same group, using a similar experimental approach, obtained similar results (Gujic et al. 2007). However, in the quoted studies only blood pressure and HR were assessed, while other important cardiovascular modulators (i.e., pre-load, inotropism, after-load) were not gathered.

In a recent paper from our lab, Mulliri et al. (2019) showed the effect on central hemodynamics of a hypoxic dynamic exercise sessions at two different levels of fraction of inspired oxygen (13.5% and 15.5% of FiO_2_) during a subsequent PEMI protocol conducted in normoxia. Authors found a reduction in the responses of SV and ventricular filling rate (VFR; a measure of venous return/ventricular diastolic function) after hypoxic exercise. Moreover, an increase in systemic vascular resistance (SVR) was detected, and this allowed to maintain MAP. It was assumed that this hemodynamic scenario was the consequence of a NO-mediated venodilation induced by exercise in hypoxia, which reduced venous return and cardiac preload, thus precluding the possibility to recruit the Frank-Starling mechanism. However, neural reflex (likely the baroreflex) successfully defended MAP on the face of SV reduction.

In the last years, exercise in hypoxia has been proposed as a potential tool for training and therapeutic purposes (Millet et al. 2010, 2016; Wilber 2001). However, hemodynamic consequences and potential risks and benefits of exercise in hypoxia has not been completely elucidated yet (Dempsey and Morgan 2015). For instance, in patients with coronary artery disease a significant decrease in exercise-induced coronary reserve has been reported already at 2500 m altitude (Wyss et al. 2003). Furthermore, mild hypoxia has been reported to cause cognitive impairment in aircraft pilots (Bouak et al. 2018). Therefore, further studies are needed to better understand the physiological and hemodynamic changes that occur when a hypoxic stimulus is applied during exercise. Specifically, considering that the few studies which dealt with the consequences of hypoxia on the cardiovascular regulation took into account only few variables (blood pressure and HR) (Gujic et al. 2007; Houssiere et al. 2005) or were conducted after exercise in hypoxia (Mulliri et al. 2019), it should be advisable to conduct a study which takes into account all the hemodynamic modulators (pre-load, inotropism, after-load, chronotropism) during hypoxic exercise. This would help in better clarify the effect of hypoxic exercise on the cardiovascular reflexes and regulation.

Starting from the above considerations, the aim of the present study was to further investigate the effect of hypoxic dynamic exercise on central hemodynamics. In particular, we were interested in discovering the cardiovascular consequences of brief dynamic exercise bouts (cycling) in hypoxia during the metaboreflex elicited by the PEMI method. We hypothesised that this condition led to results similar to those described when the metaboreflex was activated in normoxia after dynamic exercise in hypoxia. We hypothesised that similarly to what observed when the metaboreflex was activated after hypoxia, PEMI during hypoxia reduced the SV response because of a reduction in VFR.

## Methods

### Participants

Eleven healthy and recreationally active Caucasian males aged 22–46 years were recruited to participate in the study. All subjects were regularly involved in leisure-time sports activities at least three times/week. Their average values ± standard deviation (SD) of age, body mass, and height were 32.7 ± 7.2 years, 71.8 ± 10.3 kg, and 174.5 ± 5.0 cm, respectively. All participants underwent a preliminary medical examination to assess their health status. None of them suffered from cardiovascular or respiratory diseases or were on medication at the time of the experiment. All the subjects were non-smokers and abstained from drinking alcohol or coffee for at least 24 h before scheduled tests.

The study was conducted according to the Declaration of Helsinki and was approved by the ethics committee of the University of Cagliari. All the participants signed written informed consent before the beginning of the study.

### Experimental protocol

*Preliminary test *Each participant underwent a preliminary cardiopulmonary exercise stress test (CPET) on an electromagnetically braked cycle-ergometer (CUSTO Med, Ottobrunn, Germany). Oxygen uptake ($\dot{V}$O_2_), carbon dioxide production ($\dot{V}$CO_2_), and *V*_E_ were assessed with a gas analyser (VO2000, MedGraphics St. Paul, MN, USA) calibrated immediately before each test. The exercise protocol was incremental and it consisted of a linear increase of workload (30 W min^−1^), starting at 30 W, keeping a pedalling frequency of 60 rpm until exhaustion, which was considered as the point at which the subject was unable to maintain a pedalling rate of at least 50 rpm. Anaerobic threshold (AT), maximum workload (*W*_max_), and maximum oxygen uptake ($\dot{V}$O_2max_) were gathered. The following criteria were employed to consider $\dot{V}$O_2max_ achievement: a plateau in $\dot{V}$O_2_ despite increasing workload (< 80 ml min^−1^); (2) respiratory exchange ratio (RER) above 1.10; and (3) HR ± 10 beats min^−1^ of predicted maximum HR calculated as 220-age (Howley et al. 1995). AT was calculated using the *V*-slope method, which detects AT using a computerised regression analysis of the slope of $\dot{V}$CO_2_ plotted as a function of $\dot{V}$O_2_ (Beaver et al. 1986). During the preliminary test, participants familiarised with the laboratory members and equipment, allowing habituation to the environment and the ergometer that was employed in the successive experimental sessions.

*Test to study the metaboreflex during normoxia and hypoxia *After the preliminary test (interval 4–7 days), subjects underwent randomly assigned two tests to study the metaboreflex in two different conditions: one test was conducted in normoxia (NORMO) and one in normobaric hypoxia (HYPO). Test NORMO and HYPO were separated by at least 7 days (interval 7–10 days). In detail, during both NORMO and HYPO sessions, participants were connected by a mask to a hypoxic gas generator (Everest Summit II Generator, Hypoxico, New York, USA). This device separates nitrogen from oxygen thanks to a molecular sieve system that uses zeolites and provides a gas mixture with a reduced oxygen content that can be regulated to reach a minimum FiO_2_ of 12.5%. The latter corresponds approximately to the partial pressure of oxygen at 4000 m of altitude. A gas mixture with a FiO_2_ of 13.5% (corresponding to an altitude of about 3500 m) and of 21% were delivered during the HYPO and the NORMO test, respectively. Subjects were blinded about the actual content of oxygen they were breathing, which was constantly checked by an operator by means of oxygen analyser provided with the device (Maxtec, Handi+, Salt Lake City, UT, USA). A similar experimental setting was already employed in our lab in a recent investigation (Mulliri et al. 2019).

During both NORMO and HYPO tests, the subject performed randomly assigned two exercise tests pedalling on the same cycle-ergometer utilised for the CPET. The two tests were almost identical, with the exception of the first 3 minutes of the recovery phase. In detail, the subject in study sat on the cycle-ergometer for 3 min of rest; then, he pedalled for 3 min against a workload corresponding to the 30% of the *W*_max_ reached during the CPET. The recovery phase could be either PEMI or control exercise recovery (CER). In detail, during the PEMI test, immediately after the cessation of exercise, a cuff previously applied to the subject' thigh was inflated (in less than 3 s) to a pressure of 50 mmHg above the blood pressure measured at the third minute of exercise. The cuff was kept inflated for 3 min and then removed. Then, a further period of 3-min recovery was allowed. This procedure has been several times employed to elicit the metaboreflex (Crisafulli 2017; Crisafulli et al. 2008; Scott et al. 2002). Indeed, the compression applied after exercise induces a temporary arterial and venous occlusion that traps muscle metabolites produced during exercise thereby stimulating the metaboreflex and, at the same time, excluding the influence of mechanoreflex and central command (Crisafulli et al. 2008; Scott et al. 2002). The PEMI manoeuvre has been demonstrated to be effective in evoking changes in cardiac preload, afterload, and inotropism in healthy humans as well as in patients suffering from various cardiovascular diseases (Crisafulli 2017; Magnani et al. 2018; Marongiu et al. 2013; Milia et al. 2015; Mulliri et al. 2016; Roberto et al. 2017).

**Fig. 1:**
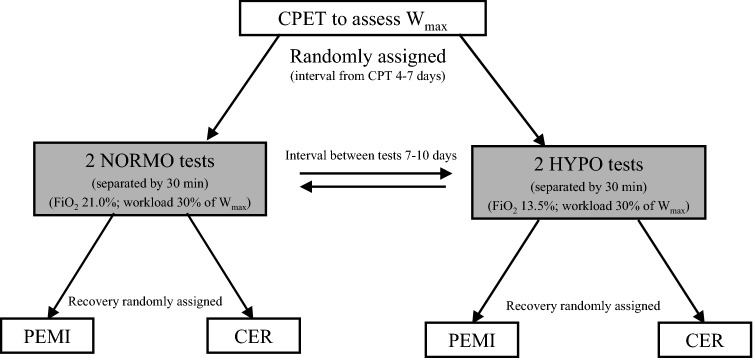
Study design. After the cardiopulmonary test (CPET, interval 4–7 days), participants underwent in separate days (interval 7–10 days), two randomly assigned exercise sessions in nomoxia (NORMO) or hypoxia (HYPO, with a FiO_2_ of 13.5%). During sessions, two exercise tests were conducted, each lasting 3 min, at a workload corresponding to 30% of the maximum previously achieved (*W*_max_) during the CPET, and separated by 30 min. After tests, subjects recovered with randomly assigned post-exercise muscle ischemia (PEMI) or control exercise recovery (CER). See text for more details

The whole PEMI session lasted 12 min in total (i.e., 3 min of rest, 3 min of exercise, 3 min of PEMI, and 3 min of further recovery). The CER session, which had the same duration (i.e., 12 min), was similar to the PEMI session, but the recovery period after exercise was conducted for 6 min without applying any occlusion on the exercised limb. Similar protocols (i.e., 3 min of rest, 3 min of exercise, 3 min of PEMI, and 3 min of further recovery) were employed in previous investigations conducted in our lab to elicit the metaboreflex by PEMI with different muscle groups (Crisafulli 2017). Furthermore, the same protocol was recently employed to study the metaboreflex in normoxia after bouts of exercise in hypoxia (Mulliri et al. 2019). PEMI and CER sessions were separated by a recovery of at least 30 min. Recovery was considered complete when HR return to values not higher than 5 bpm with respect to the pre-exercise level. The design of the study is summarised in Fig. 1. As previously pointed out, PEMI and CER tests were completed in normoxia and hypoxia.

All experiments were conducted in a room at controlled temperature and humidity (22 °C, relative humidity 50%). Tests’ randomisation was obtained using an online random sequence generator (https://www.random.org/sequences/).

### Hemodynamic assessment

Throughout all sessions, hemodynamics were collected by mean of impedance cardiography (NCCOM 3, BoMed Inc., Irvine, CA). This method has been previously used in similar experimental settings (Crisafulli et al. 2008; Mulliri et al. 2019). The impedance method allows to detect SV from changes in the thoracic impedance (*Z*_0_) measured while a low-amplitude alternate electrical current is applied to the thorax. Since electricity follows the ways of less impedance, the electrical current mainly flows along the great vessels in the mediastinum (aorta, superior, and inferior vena cava) so that the volume of blood inside the aorta is the major determinant of *Z*_0_. It follows that changes in *Z*_0_ reflect changes in blood volume inside the aorta, which in turn depends on SV. Standard formulas can be employed to derive SV from *Z*_0_ changes.

In detail, analog impedance and ECG traces were converted in digital signal and stored with a dedicated digital recorder (ADInstruments, PowerLab 8sp, Castle Hill, Australia) at a sampling rate of 500 Hz. ECG, *Z*_0_ and its first derivative (d*Z*/d*t*) were analysed offline and used to compute the pre-ejection period (PEP), the ventricular ejection time (VET), and the HR as the reciprocal of *R*–*R* intervals. For each heart beat analysed, PEP corresponded to the time interval between the onset of the QRS wave on the ECG and the beginning of the systolic deflection on the d*Z*/d*t* trace. VET was calculated as the time interval between the systolic deflection of d*Z*/d*t* and the local minimum of d*Z*/d*t* measured in the same cardiac cycle. SV was indirectly calculated from *Z*_0_, the local maximum of d*Z*/d*t*, and VET according to Bernstein's formula (1986). Diastolic time (DT) was measured as the difference between the *R*–*R* interval and the sum of PEP and VET. VFR, an index of cardiac preload and diastolic function, was obtained as the ratio of SV and DT (Gledhill et al. 1994; Marongiu et al. 2013; Milia et al. 2015). Ventricular emptying rate (VER; a measure of cardiac systolic performance) was calculated as the ratio of SV and VET (Gledhill et al. 1994; Sanna et al. 2017).

CO was obtained as the product of SV and HR, while systolic (SBP) and diastolic blood pressure (DBP) were measured by means of manual sphygmomanometer applied on the non-dominant arm. To avoid any operator-dependent bias, the same physician took care of the blood pressure measurement throughout all the experimental sessions. MAP was derived from SBP and DBP according to Moran's formula that corrects MAP measure considering changes in DT and systolic time during tachycardia (Sainas et al. 2016). Finally, SVR was indirectly obtained from the ratio of MAP and CO multiplied by 80, a conversion factor applied to correctly represent SVR as standard resistance units.

To confirm that the hypoxic stimulus was effective, peripheral blood O_2_ saturation (SO_2_) was continuously measured through finger pulse oxymetry (Nonin, SenSmart X-100, Plymouth, MN, USA). Moreover, cerebral tissue oxygenation (Cox) was assessed using near-infrared spectroscopy (NIRS) (Nonin, SenSmart X-100, Plymouth, MN, USA). One NIRS probe was positioned on the left side of the forehead over the ipsilateral eyebrow. The sensor was taped and covered with a headband to keep the probe in a fixed position and prevent outer light from interfering. The operator that instrumented the subjects for SO_2_ and Cox verified that the headband was comfortable and did not cause any blood flow occlusion.

### Data analysis

Data are presented as mean ± SD. Changes in Cox are reported as percent variation against the baseline. All recorded data were averaged over 1 min. Differences in SO_2_ and Cox were assessed using a two-way analysis of variance (ANOVA) (factors of time and condition: NORMO and HYPO) followed by Bonferroni post hoc when appropriate. Hemodynamic values were analysed at the third minute of rest, exercise, and PEMI (when metaboreflex activity was expected to be in a steady-state). Two-way ANOVA was employed to confront hemodynamic data for the effects of test (PEMI and CER) and condition (NORMO and HYPO) followed by Bonferroni post hoc when appropriate.

To further analyse the effect of metaboreflex activity on hemodynamic variables, the PEMI minus CER difference at the third minute after exercise was evaluated for all the measurements analysed. This method allowed metaboreflex response to be assessed, i.e., the response due to the metaboreflex activity (Crisafulli et al. 2013; Mulliri et al. 2016). Paired sample *t* test was used to detect changes in metaboreflex response between NORMO and HYPO. Statistical analysis was performed using commercially available software (GraphPad Prism). A *p* value < 0.05 was considered to determine statistical significance.

## Results

Results of the CPET are reported in Table 1. Table 2 depicts the values of hemodynamic variables gathered during the third minute of rest preceding the PEMI and the CER tests both in normoxia and hypoxia. Statistics did not detect any test or condition effect for any of the studied variables. Similarly, Table 3 depicts that at the third minute of exercise none of the hemodynamic variables were influenced by test or condition.

**Table 1:** Mean values ± SD of metabolic data at 30% *W*_max_, at the anaerobic threshold (AT) and at maximum workload (*W*_max_) collected during cardiopulmonary stress test

	30% *W*_max_	AT	*W*_max_
Workload (*W*)	75.68 ± 12.37	140.90 ± 31.68	232.27 ± 41.25
$\dot{V}$O_2_ (ml·kg^−1^·min^−1^)	13.74 ± 3.32	21.71 ± 3.26	34.69 ± 3.70
$\dot{V}$O_2_ (ml·min^−1^)	1010 ± 394	1569 ± 420	2587 ± 466
$\dot{V}$CO_2_ (ml·min^−1^)	953 ± 339	1771 ± 440	3533 ± 616
RER	0.95 ± 0.08	1.10 ± 0.10	1.36 ± 0.08
*V*_E_ (l·min^−1^)	22.73 ± 7.81	38.82 ± 10.91	94.02 ± 21.75
HR (bpm)	113.72 ± 9.85	140.14 ± 12.03	174.17 ± 9.20

*N* = 11

**Table 2:** Hemodynamic values during the third minute of rest preceding the post-exercise muscle ischemia (PEMI) and the control exercise recovery (CER) tests conducted in two conditions: normoxia (NORMO) and hypoxia with FiO_2_ at 13.5% (HYPO)

	NORMO	HYPO	*p* value test effect	*p* value condition effect	*p* value interaction
HR (bpm)	PEMI 94.41 ± 12.76 CER 92.90 ± 11.63	PEMI 88.67 ± 8.80 CER 88.65 ± 9.73	0.816	0.134	0.820
SV (ml)	PEMI 60.45 ± 12.39 CER 63.77 ± 13.99	PEMI 60.50 ± 9.69 CER 69.10 ± 17.26	0.091	0.439	0.447
CO (l·min^−1^)	PEMI 5.64 ± 1.23 CER 5.85 ± 1.28	PEMI 5.35 ± 0.97 CER 6.14 ± 1.89	0.238	1	0.491
VFR (ml·s^−1^)	PEMI 230.52 ± 56.03 CER 246.47 ± 102.4	PEMI 195. 47 ± 59.44 CER 240.88 ± 116.96	0.253	0.447	0.581
VER (ml·s^−1^)	PEMI 263.04 ± 54.62 CER 271.77 ± 46.29	PEMI 261.09 ± 50.30 CER 286.30 ± 71.46	0.325	0.713	0.631
MAP (mmHg)	PEMI 84.69 ± 11.37 CER 88.03 ± 7.55	PEMI 82.72 ± 11.03 CER 85.45 ± 11.37	0.341	0.474	0.923
SVR (dynes·s^−1^·cm^−5^)	PEMI 1249.06 ± 259.15 CER 1249.09 ± 311.23	PEMI 1285.95 ± 348.74 CER 1178.54 ± 265.89	0.554	0.852	0.553

*N* = 11

**Table 3:** Hemodynamic values during the third minute of exercise of the post-exercise muscle ischemia (PEMI) and the control exercise recovery (CER) tests conducted in two conditions: normoxia (NORMO) and hypoxia with FiO_2_ at 13.5% (HYPO)

	NORMO	HYPO	*p* value test effect	*p* value condition effect	*p* value interaction
HR (bpm)	PEMI 146.19 ± 11.42 CER 139.58 ± 16.89	PEMI 146.08 ± 13.59 CER 144.91 ± 14.60	0.371	0.547	0.530
SV (ml)	PEMI 143.14 ± 44.95 CER 135.49 ± 54.02	PEMI 129.42 ± 47.70 CER 154.15 ± 48.97	0.566	0.868	0.279
CO (l·min^−1^)	PEMI 21.21 ± 5.87 CER 18.91 ± 7.71	PEMI 18.96 ± 7.51 CER 22.72 ± 6.77	0.731	0.713	0.159
VFR (ml·s^−1^)	PEMI 1180.72 ± 546.28 CER 978.12 ± 482.98	PEMI 850.27 ± 350.95 CER 1071.12 ± 464.89	0.948	0.403	0.140
VER (ml·s^−1^)	PEMI 796.75 ± 239.71 CER 760.29 ± 215.00	PEMI 774.56 ± 313.81 CER 792.75 ± 274.21	0.909	0.948	0.732
MAP (mmHg)	PEMI 102.12 ± 11.86 CER 100.30 ± 13.94	PEMI 101.21 ± 9.69 CER 103.18 ± 11.26	0.983	0.783	0.596
SVR (dynes·s^−1^·cm^−5^)	PEMI 386.16 ± 221.48 CER 412.22 ± 123.96	PEMI 473.36 ± 140.85 CER 389.72 ± 116.33	0.902	0.843	0.503

*N* = 11

**Fig. 2:**
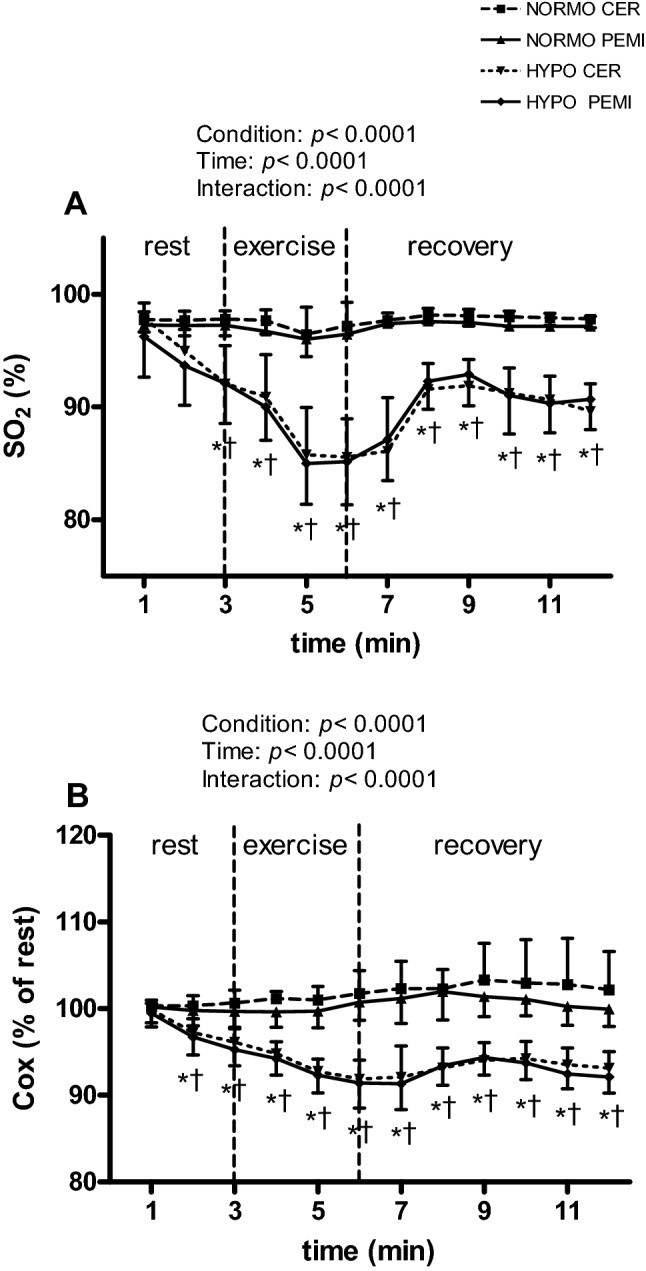
Changes in the level of peripheral blood O_2_ saturation (SO_2_, **a**) during the sessions of exercise in normoxia (NORMO CER and NORMO PEMI) and in normobaric hypoxia with a FiO_2_ of 13.5% (HIPO CER and HYPO PEMI). **b** Shows changes in cerebral oxygenation (Cox, expressed as % of baseline) during the same tests. Values are mean ± SD. *N* = 11. *P < 0.05 of HYPO CER and HYPO PEMI vs. NORMO CER; ^†^*P* < 0.05 of HYPO CER and HYPO PEMI vs. NORMO PEMI

Figure 2 shows the behaviour of SO_2_ (panel a) and Cox (panel b) during the NORMO CER, the NORMO PEMI, the HYPO CER, and the HYPO PEMI test. There was a significant reduction in both SO_2_ and Cox during the hypoxic sessions compared to the normoxic ones. These differences started at the third minute of rest for SO_2_ and at the second minute of rest for Cox to continue throughout tests duration.

**Fig. 3:**
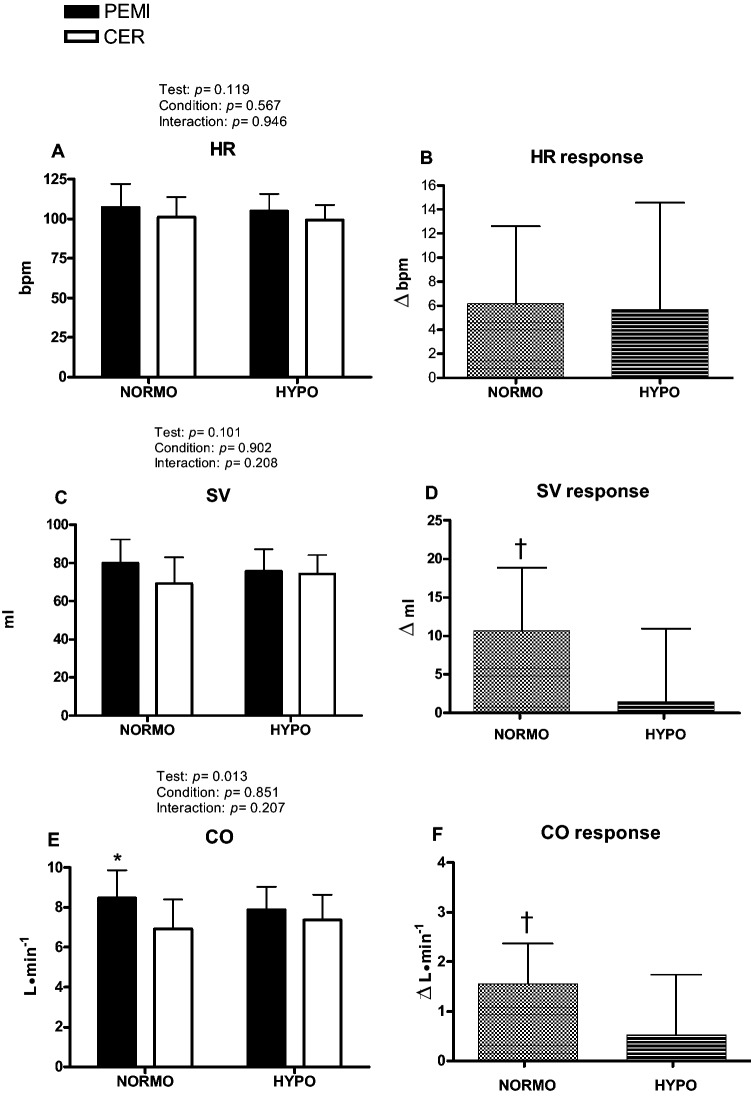
Absolute values and responses of cardiovascular variables during the post-exercise muscle ischemia (PEMI) and the control exercise recovery (CER) test, conducted after exercise in normoxia (NORMO) and in normobaric hypoxia with a FiO_2_ of 13.5% (HYPO). *HR* heart rate (**a**, **b**), *SV* stroke volume (**c**, **d**), and *CO* cardiac output (**e**, **f**). Responses were calculated as the difference between the PEMI and the CER tests at the third minute of recovery (see text for further details). Values are mean ± SD. *N* = 11. *P < 0.05 vs. CER test. ^†^*P* < 0.05 vs. response of HYPO test

**Fig. 4:**
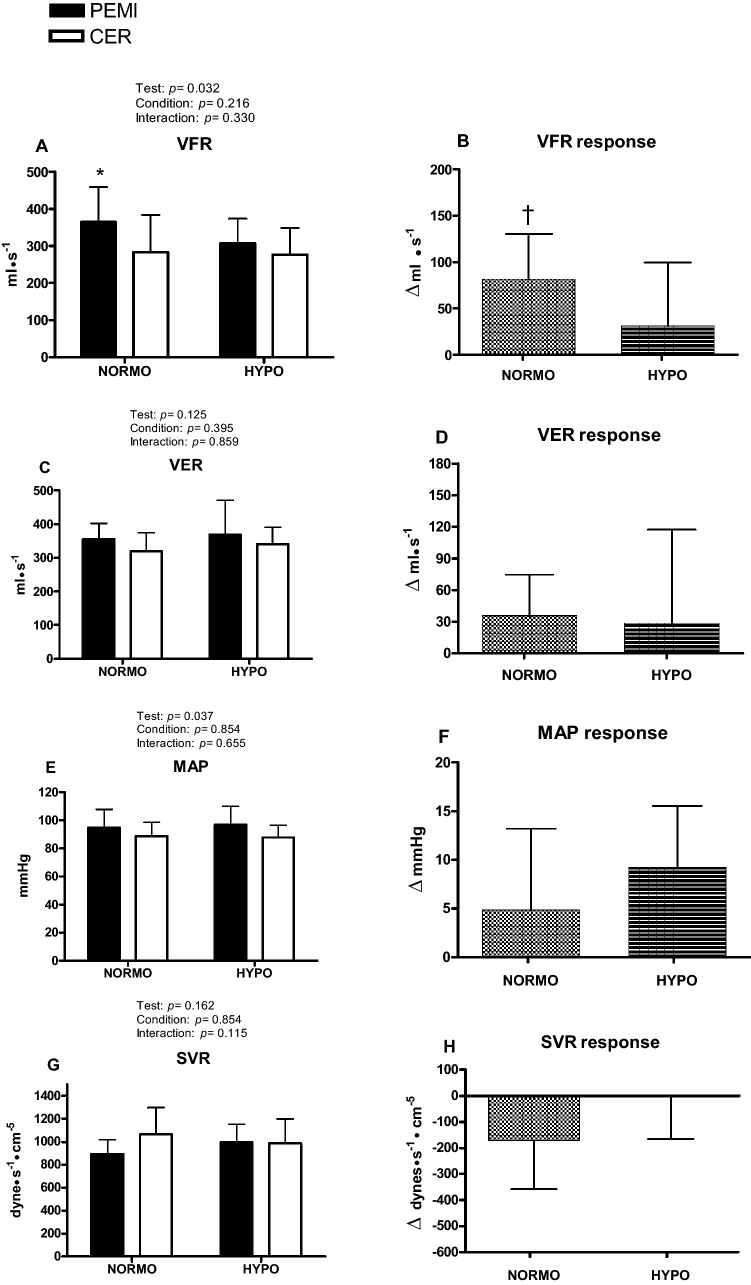
Absolute values and responses of cardiovascular variables during the post-exercise muscle ischemia (PEMI) and the control exercise recovery (CER) tests, conducted after exercise in normoxia (NORMO) and in normobaric hypoxia with a FiO_2_ of 13.5% (HYPO). *VFR* ventricular filling rate (**a**, **b**), *VER* ventricular ejection rate (**c**, **d**), *MAP* mean arterial pressure (**e**, **f**), and *SVR* systemic vascular resistance (**g**, **h**). Responses were calculated as the difference between the PEMI and the CER test at the third minute of recovery (see text for further details). Values are mean ± SD. *N* = 11. *P < 0.05 vs. CER test. ^†^*P* < 0.05 vs. response of HYPO test

Figures 3 and 4 show absolute values of hemodynamic variables measured at the third minute of recovery of the PEMI and the CER tests as well as their responses.

Figure 3 shows that exercise tests conducted in hypoxia did not affected either HR levels (panel a) or HR response to PEMI (panel b). Exercise in hypoxia did not influenced SV (panel c). However, SV response was significantly reduced by HYPO as compared to NORMO test (*p* = 0.0205; panel d). Panel e of Fig. 3 shows that there was a significant test condition for CO (*p* = 0.013). Specifically, CO was higher during the NORMO PEMI as compared to the NORMO CER test. This difference was not present between the HYPO PEMI and the HYPO CER tests. Moreover, panel f demonstrates that CO response was higher during NORMO with respect to HYPO tests (*p* = 0.0210).

Panel a of Fig. 4 shows that during the NORMO PEMI test, VFR was more elevated than during the NORMO CER test (*p* = 0.0002), and panel b shows that VFR response was significantly higher during NORMO than during HYPO sessions (*p* = 0.0034). Regarding VER, this variable was not affected by test or condition (panel c), and its response did not show any significant difference due to hypoxia (panel d). Similarly, MAP was not significantly influenced by test (panel e) and its response was similar between NORMO and HYPO conditions (panel f). Finally, panel g of Fig. 4 demonstrates that there were no differences in SVR values between NORMO and HYPO tests. Furthermore, there was no difference between NORMO and HYPO tests in SVR response (panel h), although it was close to significance (*p* = 0.075).

## Discussion

The aim of the present study was to investigate on the hemodynamic effects of contemporary normobaric hypoxia and metaboreflex activation after a brief bout of dynamic exercise. Our hypothesis was that similarly to what previously found when the metaboreflex was activated after hypoxia, the metaboreflex elicited during normobaric hypoxia resulted in a reduced capacity to increase stroke volume because of an impairment in ventricular filling rate. Results were in accordance with this hypothesis as we found a decrease in SV, VFR, and CO responses during the metaboreflex in hypoxia in comparison with the normoxic condition.

Little is known about the consequences of exercise in hypoxia in terms of cardiovascular regulation, results of the present investigation suggest that brief exercise bouts in hypoxia can reduce VFR, thereby indicating that, among hemodynamic modulators, cardiac pre-load is the variable more sensitive to hypoxic stimuli. To the best of our knowledge, the present is the first study that has demonstrated this effect during the metaboreflex stimulation. Given that exercise in hypoxia has been proposed as a tool for training and therapeutic purposes, this effect may have practical applications.

Specifically, we observed a significant difference in VFR response during hypoxic sessions as compared to the normoxic ones. Moreover, VFR was higher during the PEMI of the NORMO test than during the corresponding CER session, while this difference was not present between the HYPO tests. VFR is a measure of diastolic blood flux and its blunted response to the metaboreflex during the HYPO test suggests that a reduction in cardiac preload took place in this setting. This phenomenon may explain why SV response was also blunted as this could be related to the incapacity to effectively recruit the Frank-Starling mechanism during the metaboreflex in hypoxic condition. Furthermore, this effect was not counterbalanced by any cardiac performance enhancement, as VER, which is a measure of cardiac performance, was not different across conditions or tests.

It should also be highlighted that the lack of SV response was not compensated by any HR increment. This result is in line with other evidence indicating that HR is usually not involved in the MAP response during the metaboreflex obtained by means of PEMI, with SV and SVR acting as the main determinants of MAP adjustments (Crisafulli et al. 2009, 2015; Crisafulli 2017; Fisher et al. 2013; Iellamo et al. 1999; Nishiyasu et al. 1994). Moreover, this result is also similar to recent findings reporting that exercise under hypoxia could not elicit any HR response during PEMI (Mulliri et al. 2019).

The observation of a reduced VFR and SV responses is quite similar to what recently found in our lab when the metaboreflex was elicited in normoxia following a 10-min exercise bouts (80% of AT) at two different levels of hypoxia (15.5 and 13.5% FiO_2_). In the quoted research, the reduced VFR was explained by means of metabolite-mediated venodilation (Mulliri et al. 2019). Exercise in hypoxia actually leads to the production of a variety of vasodilating metabolites, such as NO, adenosine, and prostaglandin derived factors, and all of them potentially exert vasodilatory activity (Dinenno 2016; Marshall 2015), Hypoxia has been in the past demonstrated capable of increasing NO production by several investigations (Jia et al. 1996; Kim-Shapiro et al. 2006; Umbrello et al 2014). In this regard, it is noteworthy that oral nitrates exert a venodilator effect (Koole et al. 2000) and that the administration of NO-donors before the metaboreflex has been found able to impair cardiac pre-load by inducing venous dilation (Marongiu et al. 2013). It is possible that during the HYPO sessions there was an increase in metabolites production (with NO being the most likely candidate as a venodilator) which in turn caused a reduction in VFR and prevented the recruitment of the Frank-Starling mechanism, thus impairing the SV response. It is however to be acknowledged that this hypothesis remains speculative as we did not assess metabolite production.

Another possible explanation of the reduced VFR responses could be related to a hypoxic-induced pulmonary vasoconstriction. It has been described that even after an exposure to a short-term (10 min) hypoxia, the latter causes pulmonary arterioles constriction, thereby inducing a rise in pulmonary arterial pressure (Naeije 2010; Naeije and Chesler 2012; Naeije and Dedobbeleer 2013). This occurrence may impair left ventricular filling. In support to this hypothesis, Hsu et al. (2006) demonstrated that sildenafil, a vasodilator drug that affects pulmonary circulation, improved SV during hypoxic exercise in hypoxia but not in normoxia. Further research aimed at assessing pulmonary artery pressure should be conducted to address this point.

In the present investigation, we did not find any difference neither in SVR absolute values nor in SVR response between the NORMO and the HYPO sessions. This outcome was different with respect to the findings of our previous study, where an increase in SVR response was reported during the metaboreflex activated in normoxia after exercise in hypoxia (Mulliri et al. 2019). We postulated that this result was the consequence of the baroreflex activity, which successfully counteracted the reduced CO response by inducing arteriolar constriction, whereas after normoxia this occurrence was absent. Differently, in the present study, SVR response failed to reach statistical significance. This result could also be explained by taking into account that in the present research a shorter hypoxic stimulus was applied, as the exercise in hypoxia was conducted for 3 minutes, while in our previous investigation participants were exposed to a 10-min hypoxic stimulus during cycling at an intensity corresponding to 80% of AT. It is conceivable that a 10-min hypoxic exercise bout could have led to a more pronounced metabolic and hemodynamic challenge. Actually, the reduction in VFR response was more evident in our previous study than in the present one.

This hypothesis is further strengthened by the fact that the absolute values of HR did not change during hypoxia with respect to the normoxic condition. In this regard, it is well established that hypoxia normally leads to tachycardia (Halliwill and Minson 2002). Thus, the lack of any HR change is consistent with the concept that the hypoxic stimulus was probably mild in our experimental setting, although data from SO_2_ and Cox suggested that a hypoxic stress was actually present.

Nonetheless, the present study demonstrates that in normal individuals, a bout of hypoxic exercise even of brief duration, can substantially alter hemodynamics without affecting the blood pressure response during the metaboreflex. Moreover, the present study suggests that among the cardiovascular modulators (i.e., chronotropism, inotropism, after-load, and pre-load), cardiac pre-load is the variable more sensitive to hypoxia.

It seems well ascertained by human as well as by animal studies that in healthy individuals, the metaboreflex activation enhances cardiac performance and pre-load thereby sustaining SV (Bastos et al. 2000; Crisafulli et al. 2008; Crisafulli et al. 2009; Crisafulli et al. 2013; Crisafulli 2017; Nobrega et al. 2014; O'Leary and Augustyniak 1998; Sheriff et al. 1998; Shoemaker et al. 2005). Actually, pathological impairment in one of these hemodynamic modulators (as happens for example in heart failure, spinal cord injury, or diastolic dysfunction) blunts the SV response to metaboreflex, but usually the MAP is well preserved (Crisafulli 2017; Crisafulli et al. 2009; Milia et al. 2015; Magnani et al. 2018; Roberto et al. 2017; Sala-Mercado et al. 2006).

In the present investigation, cardiac performance did not appear to be influenced by the HYPO sessions, as the index we used (i.e., VER) did not show any difference between conditions. Therefore, the reduced SV response was likely the consequence of a reduced cardiac pre-load. According to previous evidence, pre-load seems to be increased during metaboreflex activation as a result of a sympathetic-mediated peripheral venoconstriction and splanchnic vasoconstriction, that together concur in enhancing venous return and in “centralising” blood volumes (Bastos et al. 2000; Crisafulli et al. 2009; Milia et al. 2015; Sheriff et al. 1998).

Overall, although different in the duration of the protocol (10 vs. 3 min exercise), intensity of exercise (80% AT vs. 30% *W*_max_), and experimental setting (hypoxia before vs. during the metaboreflex), results from the previous and the present study suggest that exercise in hypoxia affects the hemodynamic modulation during the metaboreflex, with the reduction in cardiac pre-load playing a pivotal role in the phenomenon.

### Limitations of our study

Some limitations of the present study should be honestly acknowledged.

Specifically, no blood samples were collected during experiments, which makes our hypothesis of a metabolite-induced venous dilating effect during exercise in hypoxia speculative at this time. Further studies comprehending blood analysis of NO by-products (nitrites, nitrates) are required to confirm if NO exerts an active role in reducing venous return.

Another limit was the lack of cardiac volumes assessment. In this regard, the addition of echocardiographic and Doppler measurements would have been useful, as this imaging technique could allow the evaluation of end diastolic volume and the indirect measure of pulmonary artery pressure. Thus, echocardiography may clarify the possible mechanisms behind the reduced SV and VFR responses that occur during the metaboreflex in hypoxia. This limitation clearly contributes to cautious interpretation of our results.

### Conclusions

Overall, the results of the present investigation supported the hypothesis that brief exercise bouts in hypoxia was capable to blunt the stroke volume response during the metaboreflex activation obtained with the post-exercise muscle ischemia method under hypoxic condition. This was the consequence of a reduced ventricular filling rate probably due to a metabolite-induced venodilation which counteracted the sympathetic-induced venoconstriction that normally occurs during the metaboreflex. Results are in good accordance with those of a recent study conducted during the metaboreflex in normoxia after exercise in hypoxia and collectively support the concept that among hemodynamic modulators, cardiac pre-load is the variable more sensitive to hypoxic stimuli. Considering that exercise in hypoxia has been proposed as a useful tool for training and therapeutic purposes, its cardiovascular effects should be further investigated to better understand physiological and hemodynamic consequences.

## Abbreviations

AT: Anaerobic threshold
CER: Control exercise-recovery
CO: Cardiac output
Cox: Cerebral tissue oxygenation
CPET: Cardiopulmonary exercise stress test
DBP: Diastolic blood pressure
DT: Diastolic time
d*Z*/d*t*: First derivative of thoracic impedance
EPR: Exercise pressor reflex
HR: Heart rate
HYPO: Tests conducted in hypoxia
MAP: Mean blood pressure
NIRS: Near infrared spectroscopy
NO: Nitric oxide
NORMO: Tests conducted in normoxia
PEMI: Post-exercise muscle ischemia
PEP: Pre-ejection period
RER: Respiratory exchange ratio
SBP: Systolic blood pressure
SD: Standard deviation
SNS: Sympathetic nervous system
SO_2_: Peripheral blood O_2_ saturation
SV: Stroke volume
SVR: Systemic vascular resistance
*V*_E_: Pulmonary ventilation
VER: Mean ventricular ejection rate
VET: Left ventricular ejection time
VFR: Ventricular filling rate
V̇CO_2_: Carbon dioxide production
V̇O_2_: Oxygen uptake
V̇O_2max_: Maximum oxygen uptake
*W*_max_: Maximum workload
*Z*_0_: Thoracic impedance
